# Supplementary material for: Geoelectrochemistry-driven alteration of amino acids to derivative organics in carbonaceous chondrite parent bodies
Source: Nat Commun. 2022 Aug 19;13:4893. doi: 10.1038/s41467-022-32596-3 (PMC9391434; doi:10.1038/s41467-022-32596-3)
Supplement: Supplementary file 1 — Supplementary Information [file 41467_2022_32596_MOESM1_ESM.pdf]

Supplementary information for

Geoelectrochemistry-driven alteration of amino acids to derivative organics in carbonaceous chondrite parent bodies

Yamei Li[1]*, Norio Kitadai[1,2], Yasuhito Sekine[1,3], Hiroyuki Kurokawa[1], Yuko Nakano,[1] Kristin Johnson-Finn[1,4]

*Corresponding author. Email: yamei.li@elsi.jp

This PDF file includes:

Supplementary Fig. 1. A photograph of the electrochemical reactor. 3
Supplementary Fig. 2. XRD patterns of iron sulfide (a) and nickel sulfide (b) synthesized by precipitation. 4
Supplementary Fig. 3. Retention time (a) and calibration curves (b-e) for ammonia and aliphatic amines by the LC-fluorescence detector system.. 5
Supplementary Fig. 4. Retention time (a) and calibration curves (b-d) for amino acids by the LC-fluorescence detector system. 6
Supplementary Fig. 5. Retention time (a) and calibration curves (b) for carboxylic acids and α-hydroxy acids by the ion chromatograph-ionic conductivity detector system. 7
Supplementary Fig. 6. Fluorescence chromatograms of solution products after electrolysis of glycine (a, b), alanine (c, d), and valine (e, f)... 8
Supplementary Fig. 7. IC chromatograms of solution products after electrolysis of glycine (a, d), alanine (b, e) and valine (c, f) 9
Supplementary Fig. 8. a: ^{1}H-NMR spectra of solution products after electrolysis of glycine on FeS at potentials of –0.5 ~ −1.0 V. b: ^{1}H-NMR spectra of authentic standards. 10
Supplementary Fig. 9. a: ^{1}H-NMR spectra of solution products after electrolysis of glycine on NiS at potentials of –0.5 ~ −1.0 V. b: ^{1}H-NMR spectra of authentic standards. 11
Supplementary Fig. 10. a: ^{1}H-NMR spectra of solution products after electrolysis of alanine on FeS at potentials of –0.5 ~ −1.0 V. b: ^{1}H-NMR spectra of authentic standards. 12
Supplementary Fig. 11. a: ^{1}H-NMR spectra of solution products after electrolysis of alanine on NiS at potentials of –0.5 ~ −1.0 V. b: ^{1}H-NMR spectra of authentic standards. 13
Supplementary Fig. 12. a: ^{1}H-NMR spectra of solution products after electrolysis of valine on FeS at potentials of –0.5 ~ −1.0 V. b: ^{1}H-NMR spectra of authentic standards. 14
Supplementary Fig. 13. a: ^{1}H-NMR spectra of solution products after electrolysis of valine on NiS at potentials of –0.5 ~ −1.0 V. b: ^{1}H-NMR spectra of authentic standards. 15

Supplementary Fig. 14. Molar concentrations of methylamine (a) and glycolate (b) generated after glycine electrolysis as a function of initial glycine concentration (0.2 ~ 20 mM) at potential of –0.9 V by using FeS or NiS as the catalyst. 16
Supplementary Fig. 15. Product distribution of glycine electrolysis at –0.9 V on NiS for 14-day reaction in a phosphate buffered (pH 7) or bicarbonate buffered (pH 8.2) electrolyte solution... 17
Supplementary Fig. 16. a) ^{1}H-NMR spectrum of products generated by electrolysis of 5 mM CN^- in phosphate buffer (pH=7) on NiS for two weeks. b) ^{1}H-NMR spectra of authentic standards.. 18
Supplementary Fig. 17. Retention time (a) and calibration curves (b) for L- and D-amino acids detected by UV-HPLC system using a chiral column. 19
Supplementary Fig. 18. Molar concentrations of primary amines generated in the electrochemical decomposition system of glycine, alanine, and valine, under –0.9 V on FeS (black) and NiS (red) at the initial amino acids concentration of 2 mM. 20
Supplementary Fig. 19 ^{1}H-NMR spectra of starting compounds (glycine, alanine, valine) and phosphate buffer.. 21
Supplementary Fig. 20 IC (a) and fluorescence (b) chromatograms of a control sample without adding any of the amino acid. 22
Supplementary Fig. 21 IC (a) and fluorescence (b) chromatograms of a control sample without using any mineral catalyst... 23
Supplementary Fig. 22 Fluorescence chromatograms of samples generated by electrolysis of aspartic acid. 24
Supplementary Fig. 23 Fluorescence chromatograms of samples generated by electrolysis of glutamic acid.. 25
Supplementary Fig. 24 IC chromatograms of samples generated by electrolysis of aspartic acid. 26
Supplementary Fig. 25 IC chromatograms of samples generated by electrolysis of glutamic acid. 27
Supplementary Fig. 26 Reaction pathways during electrolysis of aspartate and glutamate. The reaction steps are deduced based on the product analyses in Supplementary Figs. 22-25. 28
Supplementary Table 1. Summary of the product concentrations after two-week electrolysis of glycine using either FeS or NiS as the catalyst. 29
Supplementary Table 2. Summary of the product concentrations after two-week electrolysis of alanine using either FeS or NiS as the catalyst. 30
Supplementary Table 3. Summary of the product concentrations after two-week electrolysis of valine using either FeS or NiS as the catalyst. 31
Supplementary Table 4. Abundances of amino acids in various CM and CR chondrites. 32
Supplementary Table 5. Abundances of amines in various CM and CR chondrites. 32
Supplementary Table 6. Abundances of hydroxy acids in various CM and CR chondrites. 33
Supplementary Table 7. Abundance ratios of methylamine/glycine ([MA]/[Gly]), glycolate/glycine ([GA]/[Gly]), ethylamine/alanine ([EA]/[Ala]), lactate/alanine ([LA]/[Ala]), isobutylamine/valine ([IBA]/[Val]), 2-hydroxy-3-methylbutyrate/valine ([HMA]/[Val]) in different CR chondrites based on the data listed in Supplementary Tables 4-6. 33
Supplementary Table 8. Molar ratios of glycolate/methylamine, lactate/ethylamine, and 2-hydroxy-3-methylbutyrate/isobutylamine ([HMA]/[IBA]) in five different CR chondrites (GRO 95577 (CR2.0), MIL 090001 (CR2.4), GRA 95229 (CR2.7), MIL090657 (CR2.7), and QUE 99177 (CR2.8)) calculated based on reported abundances in literatures as tabulated in Supplementary Tables 5-6. 34

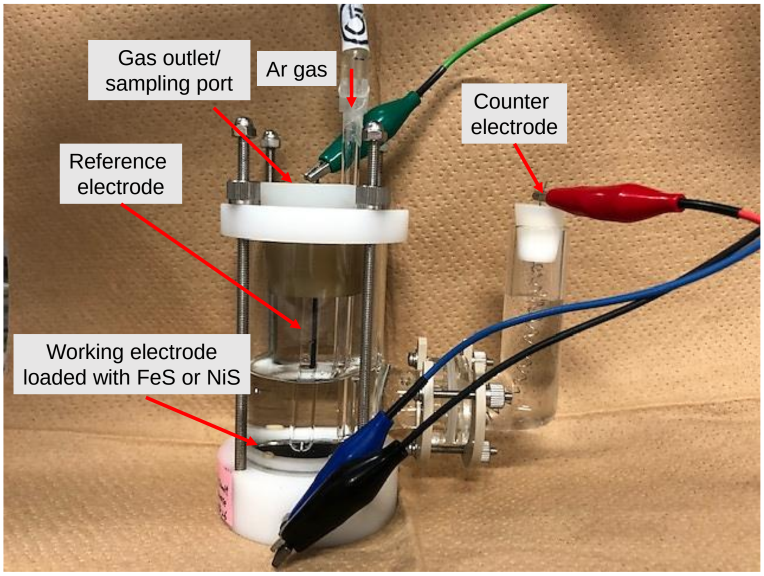

Supplementary Fig. 1. A photograph of the electrochemical reactor.

The cell is made of a Pyrex glass tube sandwiched between a polyoxymethylene (POM) cap and basement that were tightened together with stainless screws and knurled nuts. The structure was also reported in our previous work (40). The cell has two compartments: a large working electrode side (~100 mL) and a small counter electrode side (~15 mL) that are separated by a proton exchange membrane (Nafion 117; DuPont). On the working electrode side, a titanium cylinder (purity; 99.5%) is placed at the center of the POM basement, and is coated with carbon paper (5.7 cm^2) with a silicon and POM packings. An Ag/AgCl electrode (in saturated KCl) is used as the reference electrode. On the counter side, a platinum coil is inserted into the glass tube, and is used as the counter electrode. The silicon cap has three holes for inserting a gas channeling glass tube, a reference electrode and the gas outlet/sampling port.

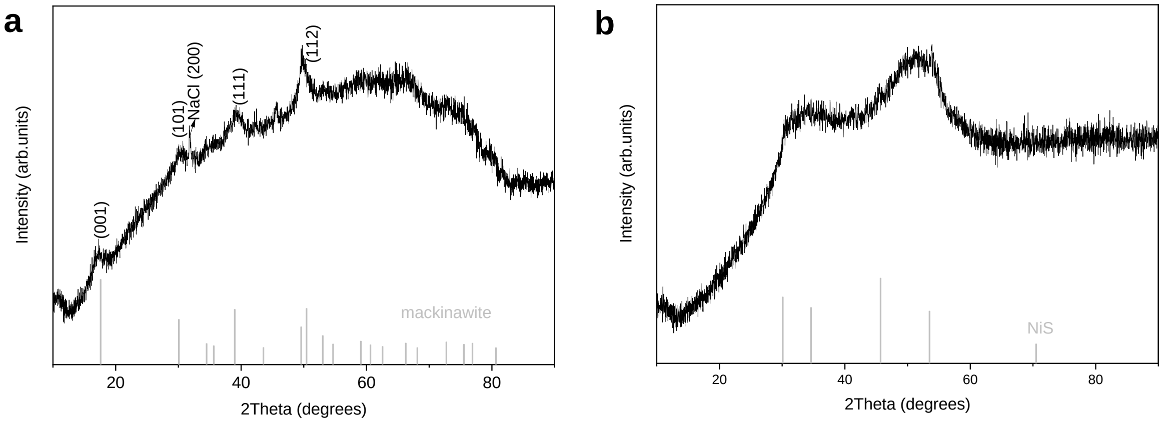

Supplementary Fig. 2. XRD patterns of iron sulfide (a) and nickel sulfide (b) synthesized by precipitation.

All measurements were conducted with 2θ ranging from 10° to 90° with a step of 0.02^o and a scan rate of 1° min^{-1}. Reference patterns were taken from the PDF (Power Diffraction File) published by the International Centre for Diffraction Data. Iron sulfide is mackinawite, and nickel sulfide is amorphous with only broad diffraction bands assignable to NiS. One sharp peak in a is assignable to NaCl.

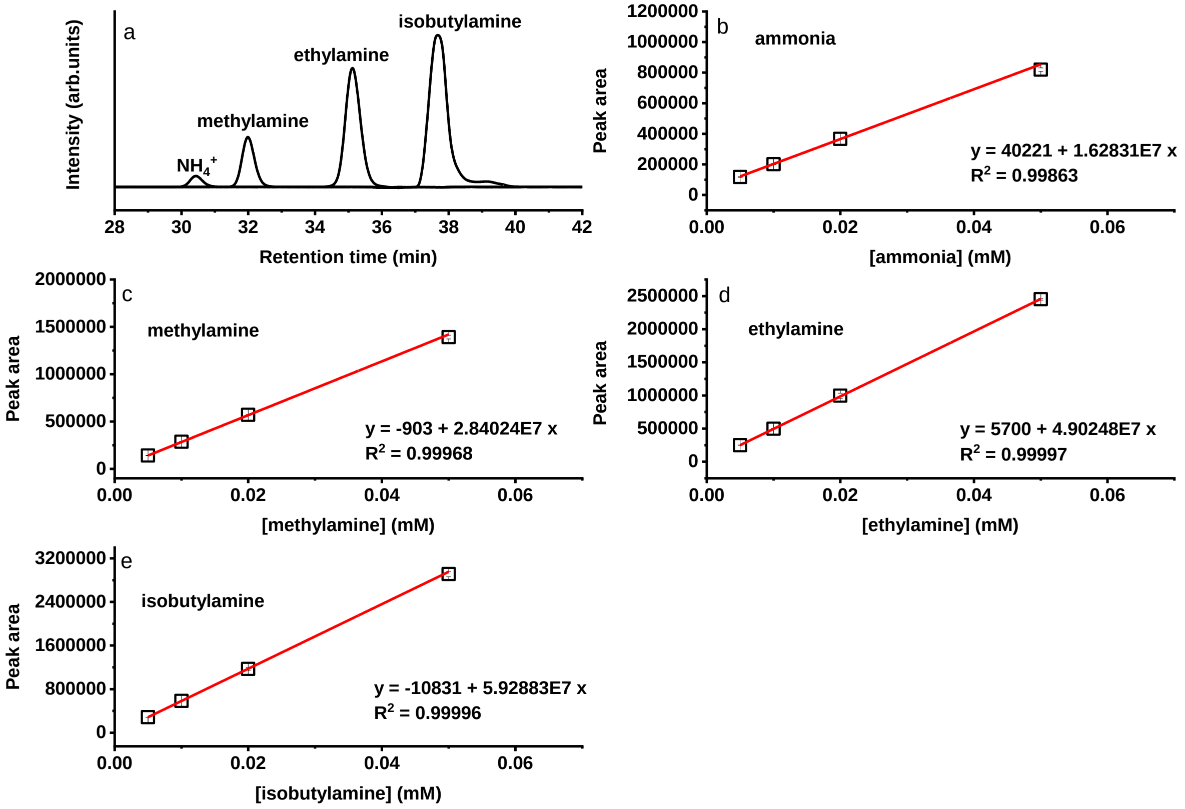

Supplementary Fig. 3. Retention time (a) and calibration curves (b-e) for ammonia and aliphatic amines by the LC-fluorescence detector system.

The error bars are the standard deviations associated with the repeated measurements.

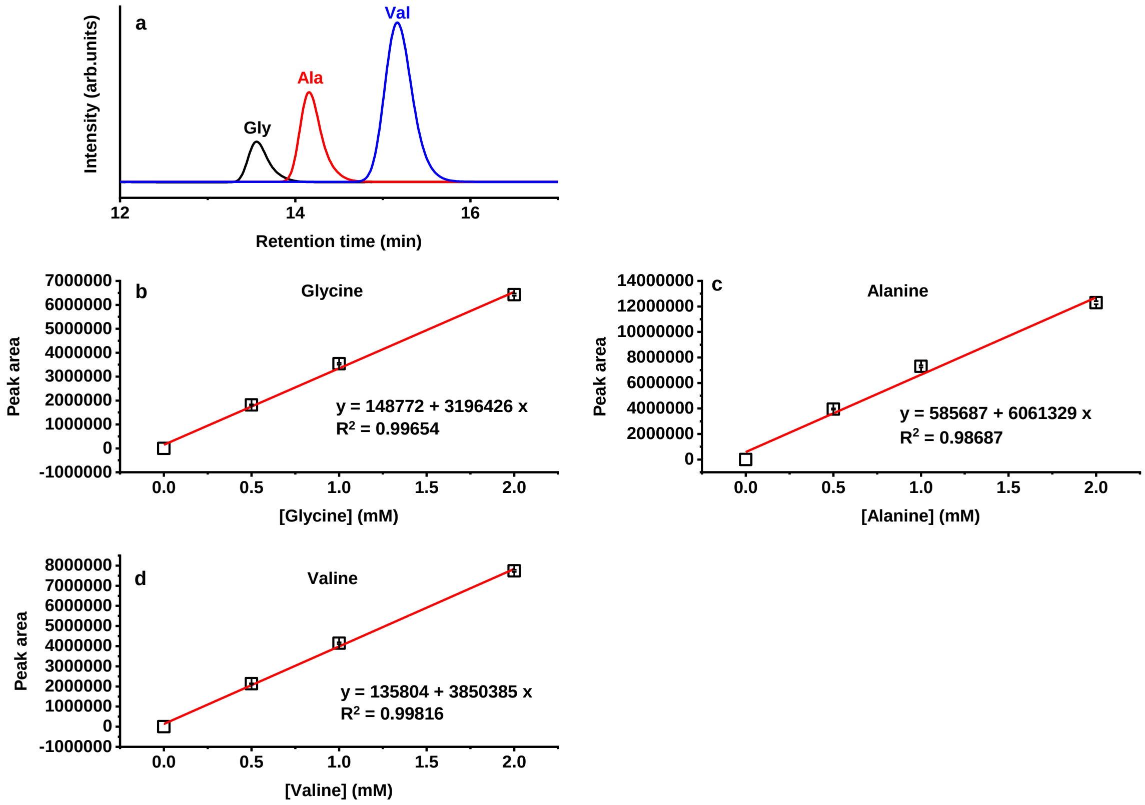

Supplementary Fig. 4. Retention time (a) and calibration curves (b-d) for amino acids by the LC-fluorescence detector system.

The error bars are the standard deviations associated with the repeated measurements. Abbreviations in a: Gly: glycine; Ala: alanine; Val: valine.

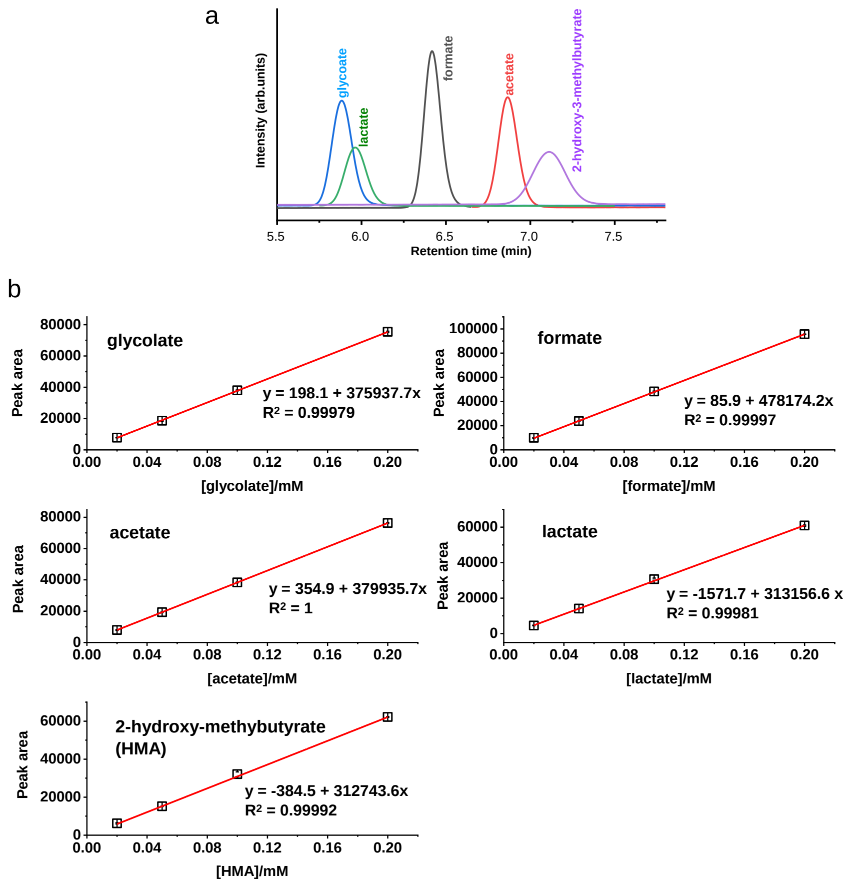

Supplementary Fig. 5. Retention time (a) and calibration curves (b) for carboxylic acids and α-hydroxy acids by the ion chromatograph-ionic conductivity detector system.

a shows the retention time of monocarboxylic acids (formic acid, acetic acid), and hydroxy acids (glycolic acid, lactic acid, 2-hydroxy-3-methylbutyric acid). The error bars are the standard deviations associated with the repeated measurements.

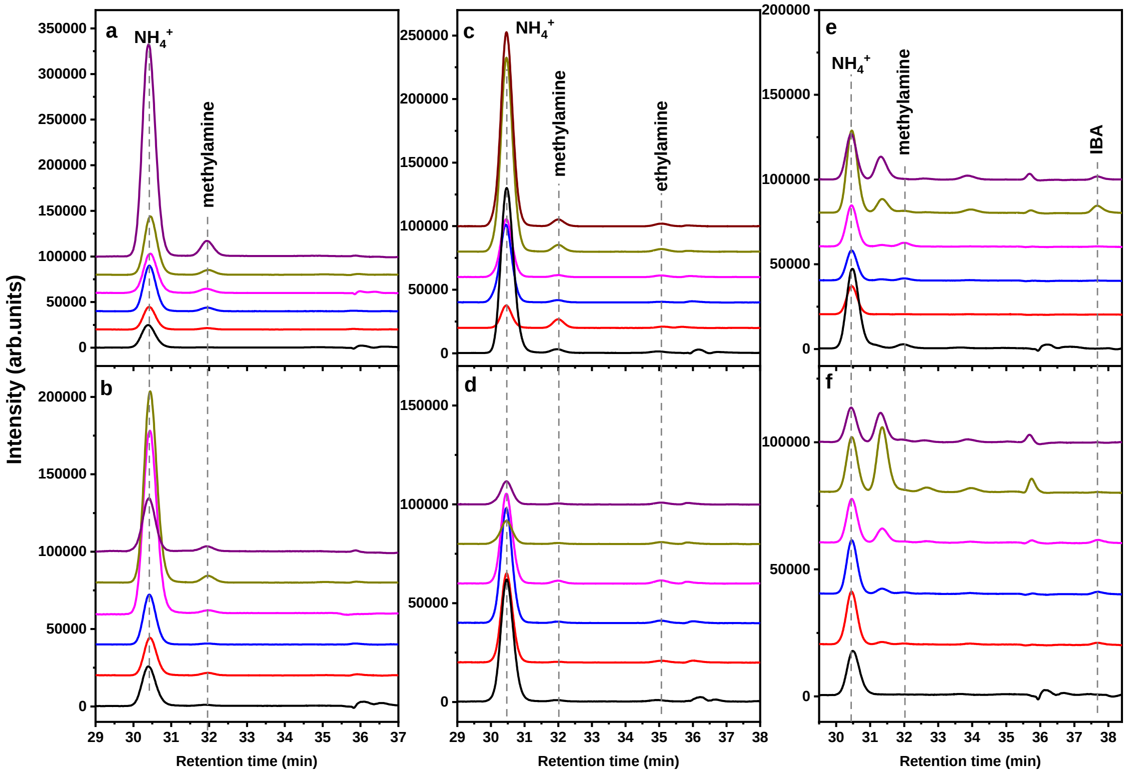

Supplementary Fig. 6. Fluorescence chromatograms of solution products after electrolysis of glycine (a, b), alanine (c, d), and valine (e, f).

Electrolysis were conducted at potentials of −0.5 V (black), −0.6 V (red), −0.7 V (blue), −0.8 V (pink), −0.9 V (brown), and −1.0 V (purple) by using FeS (a, c, and e) or NiS (b, d, and f) as the catalyst. Abbreviation: isobutylamine (IBA).

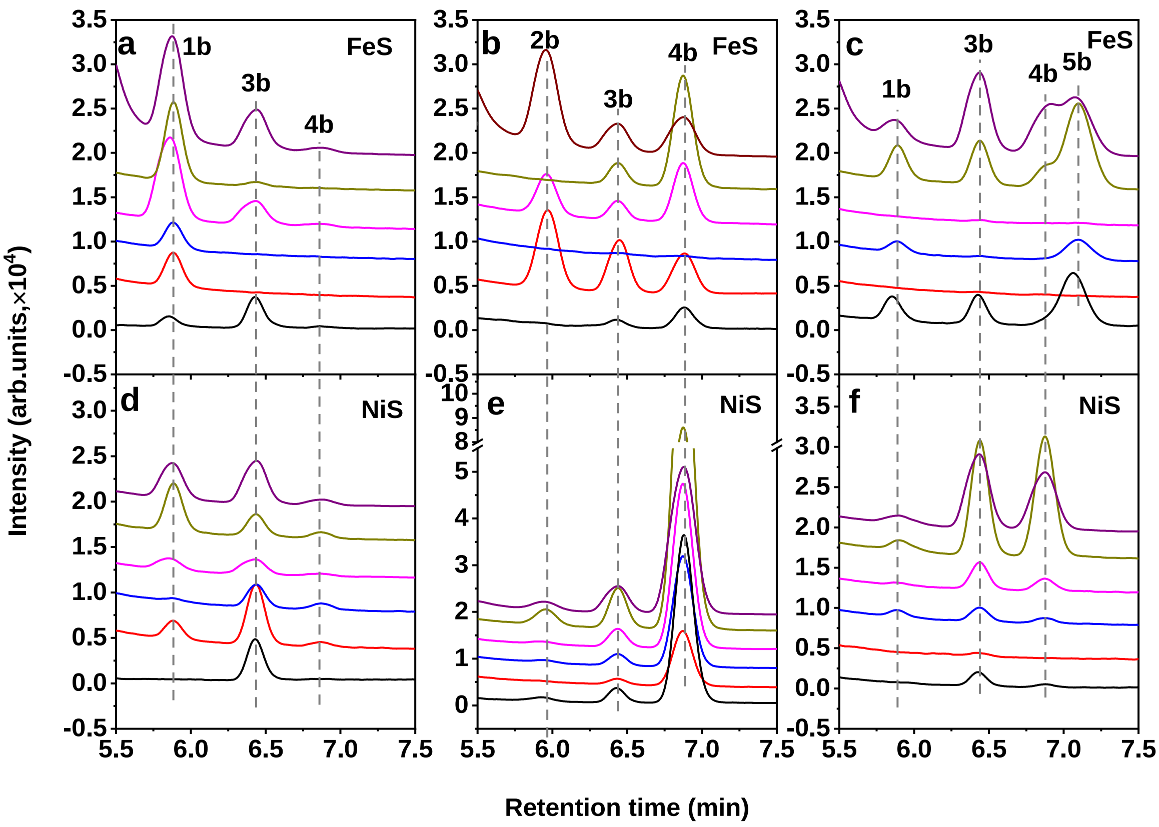

Supplementary Fig. 7. IC chromatograms of solution products after electrolysis of glycine (a, d), alanine (b, e) and valine (c, f)

Electrolysis were conducted at potentials of −0.5 V (black), −0.6 V (red), −0.7 V (blue), −0.8 V (pink), −0.9 V (brown), and −1.0 V (purple) by using FeS (upper panels) or NiS (bottom panels) as the catalyst. Peak identities: 1b) glycolate; 2b) lactate; 3b) formate; 4b) acetate; 5b) 2-hydroxy-3-methylbutyrate (HMA);

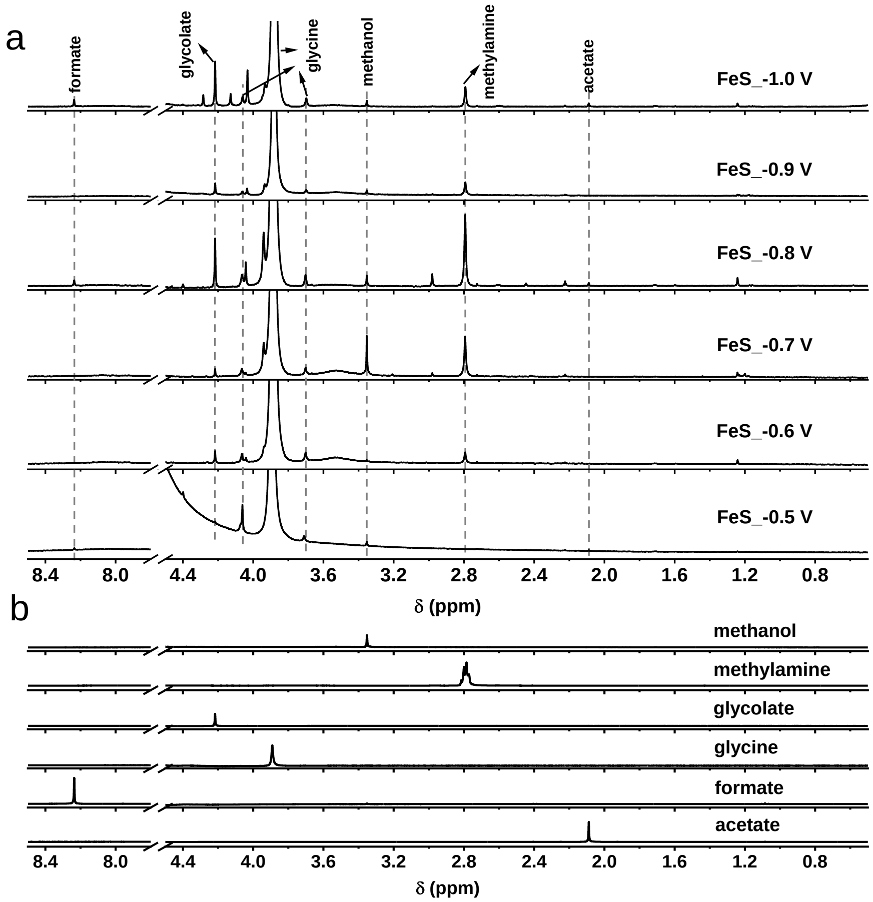

Supplementary Fig. 8. a: ^{1}H-NMR spectra of solution products after electrolysis of glycine on FeS at potentials of −0.5 ~ −1.0 V. b: ^{1}H-NMR spectra of authentic standards.

Methanol was detected under several conditions; however, it was also detected in a control where amino acids were not implemented. Therefore, methanol was not considered as a product.

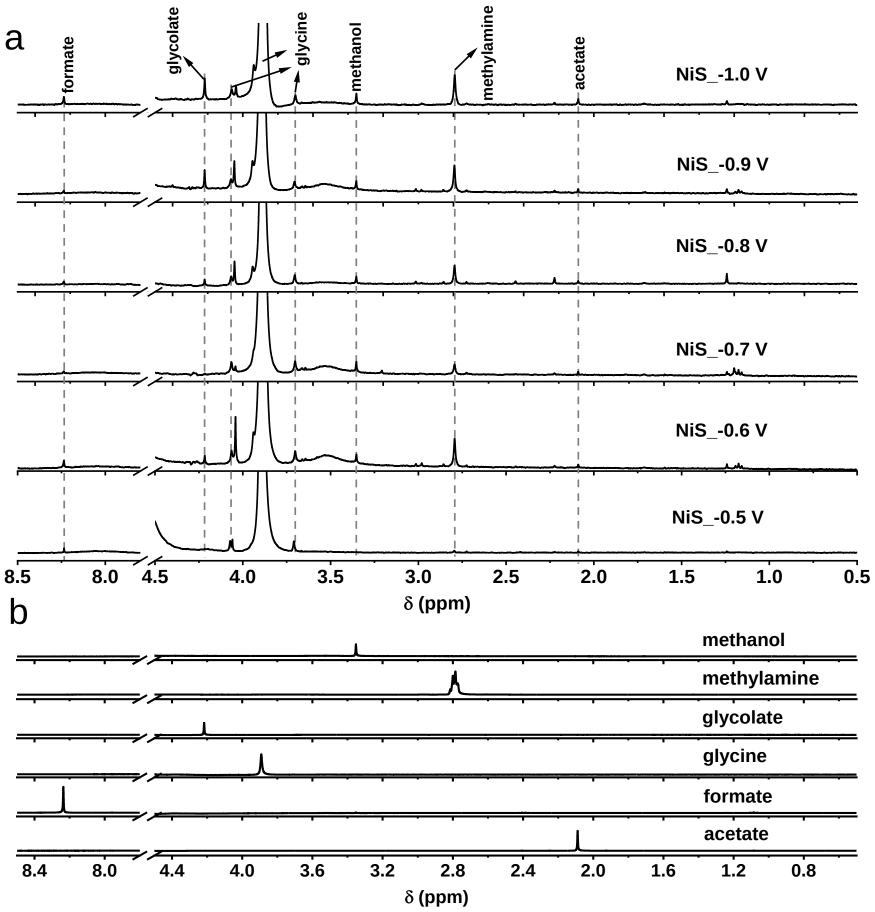

Supplementary Fig. 9. a: ^{1}H-NMR spectra of solution products after electrolysis of glycine on NiS at potentials of −0.5 ~ −1.0 V. b: ^{1}H-NMR spectra of authentic standards.

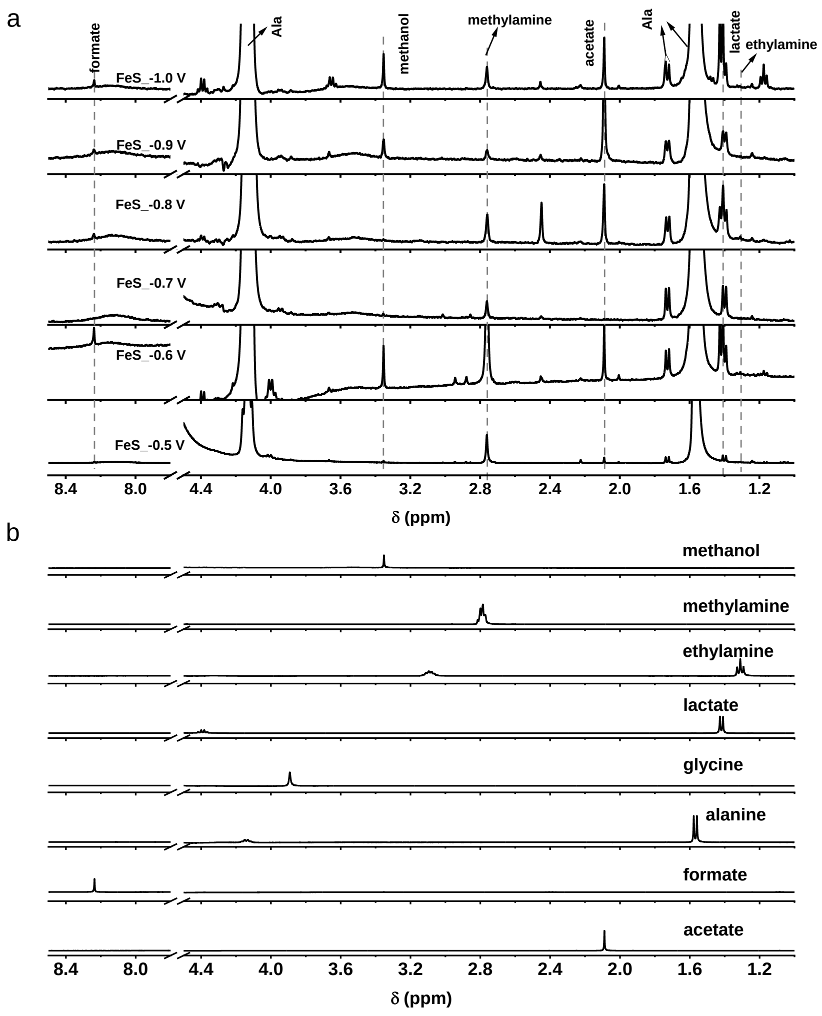

Supplementary Fig. 10. a: ^{1}H-NMR spectra of solution products after electrolysis of alanine on FeS at potentials of −0.5 ~ −1.0 V. b: ^{1}H-NMR spectra of authentic standards.

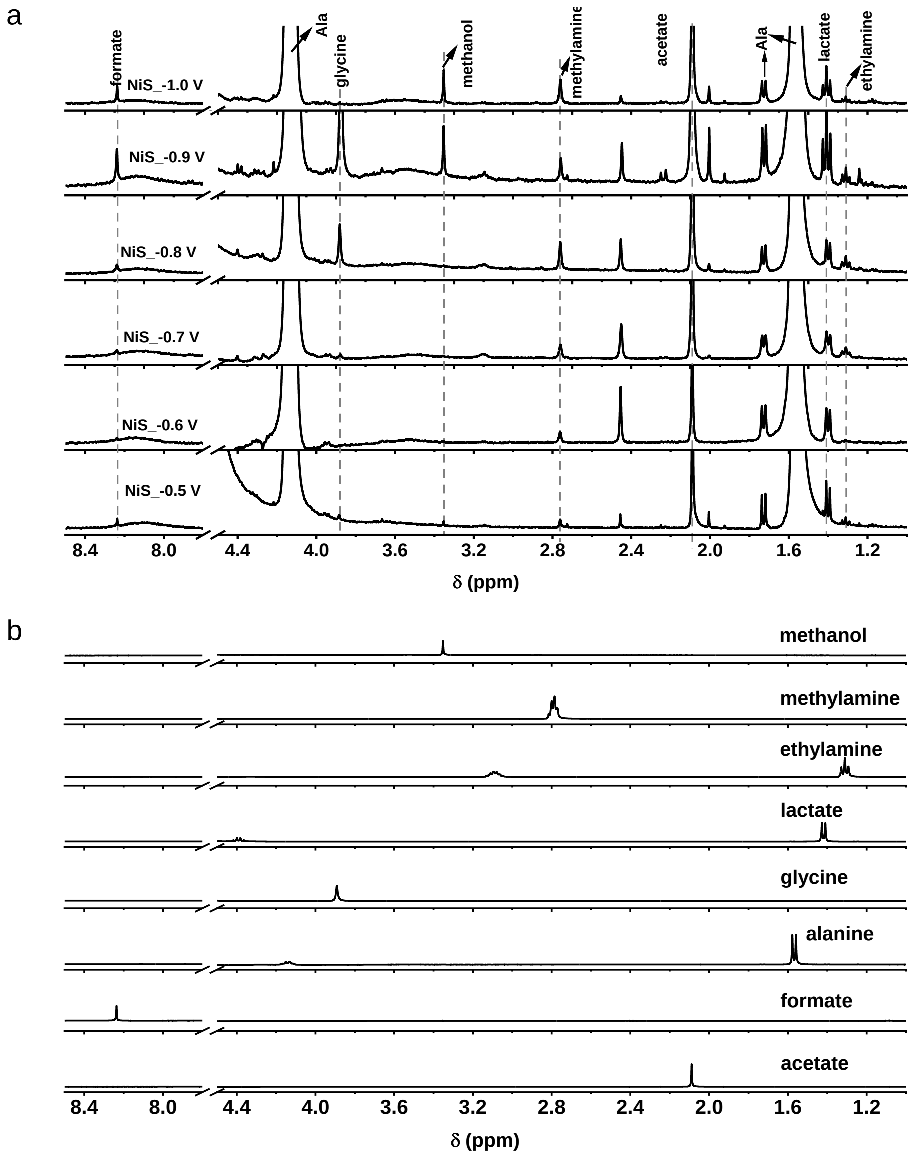

Supplementary Fig. 11. a: ^{1}H-NMR spectra of solution products after electrolysis of alanine on NiS at potentials of −0.5 ~ −1.0 V. b: ^{1}H-NMR spectra of authentic standards.

a

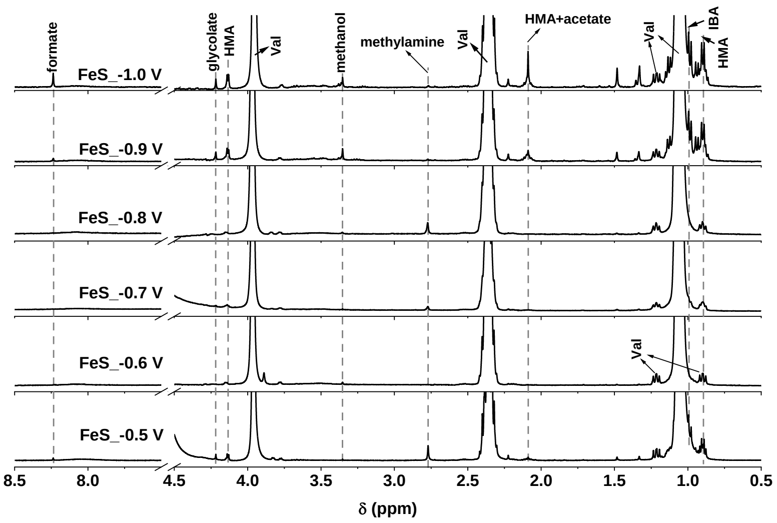

b

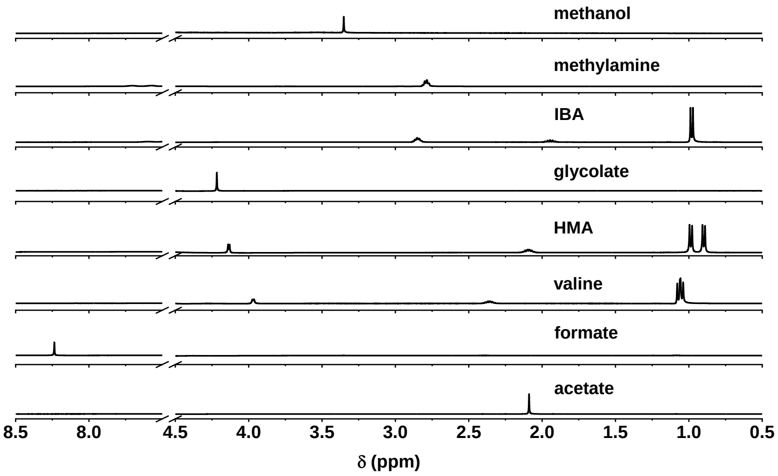

Supplementary Fig. 12. a: ^{1}H-NMR spectra of solution products after electrolysis of valine on FeS at potentials of −0.5 ~ −1.0 V. b: ^{1}H-NMR spectra of authentic standards.

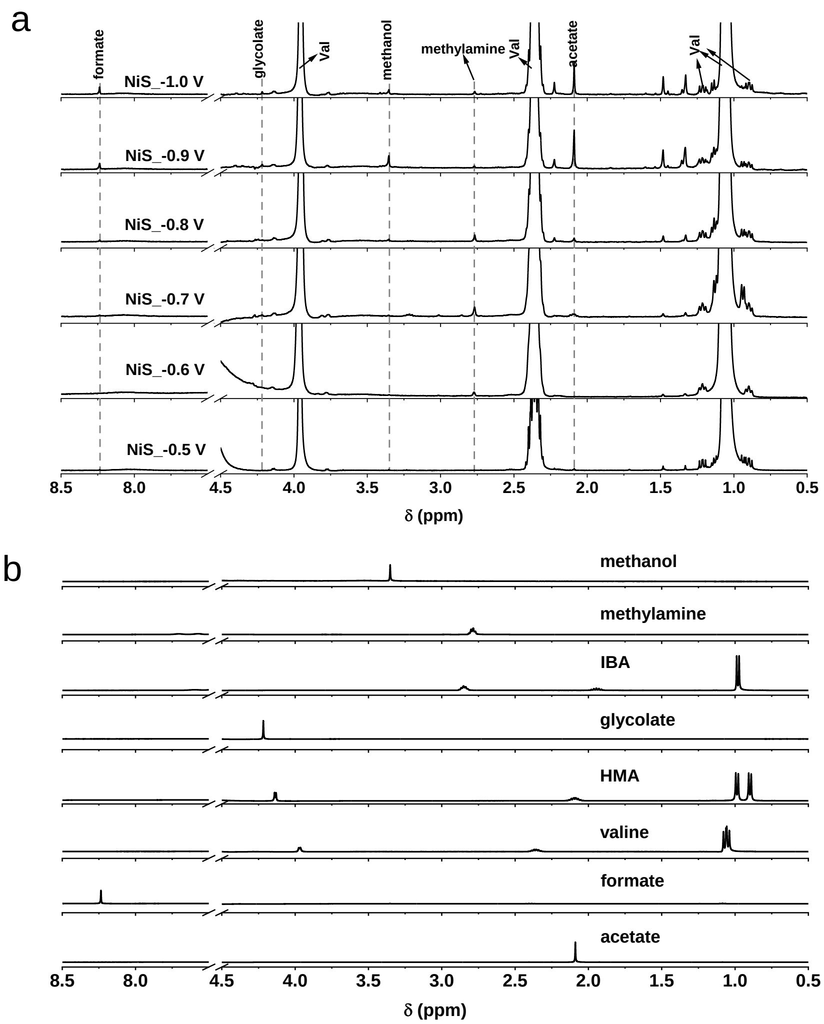

Supplementary Fig. 13. a: ^{1}H-NMR spectra of solution products after electrolysis of valine on NiS at potentials of −0.5 ~ −1.0 V. b: ^{1}H-NMR spectra of authentic standards.

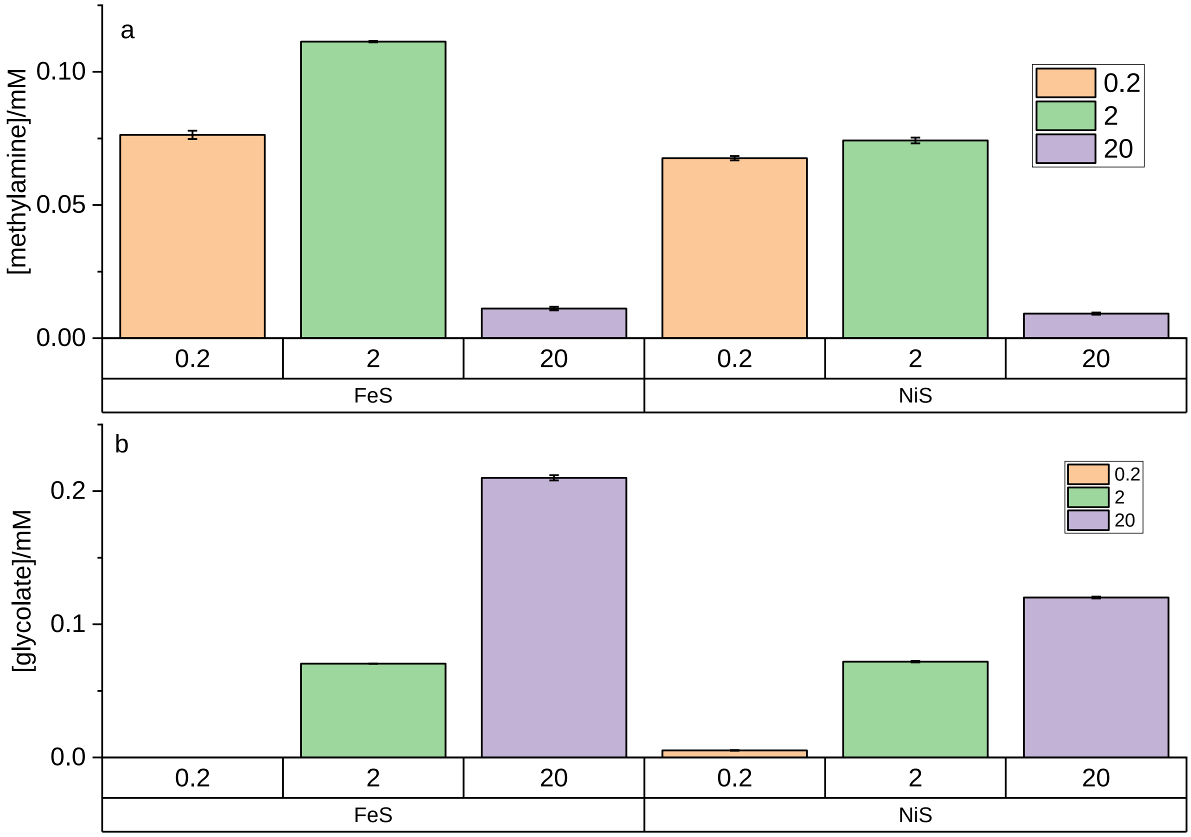

Supplementary Fig. 14. Molar concentrations of methylamine (a) and glycolate (b) generated after glycine electrolysis as a function of initial glycine concentration (0.2 ~ 20 mM) at potential of –0.9 V by using FeS or NiS as the catalyst.

The error bars are the standard deviations associated with the repeated measurements.

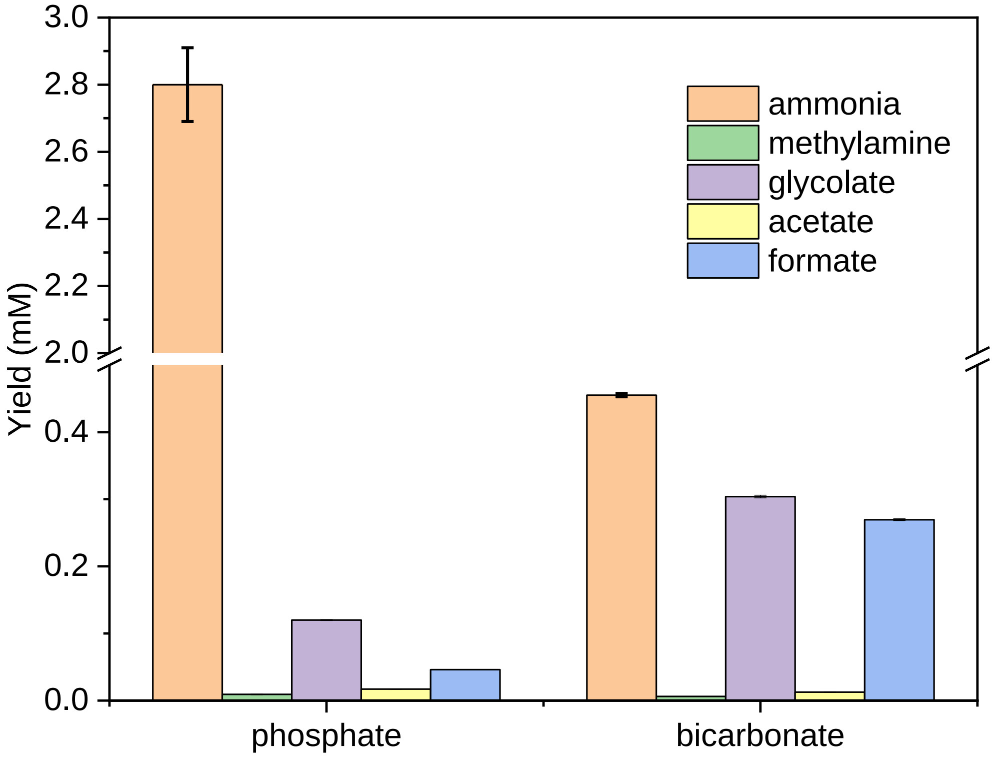

Supplementary Fig. 15. Product distribution of glycine electrolysis at –0.9 V on NiS for 14-day reaction in a phosphate buffered (pH 7) or bicarbonate buffered (pH 8.2) electrolyte solution.

The error bars are the standard deviations associated with the repeated measurements.

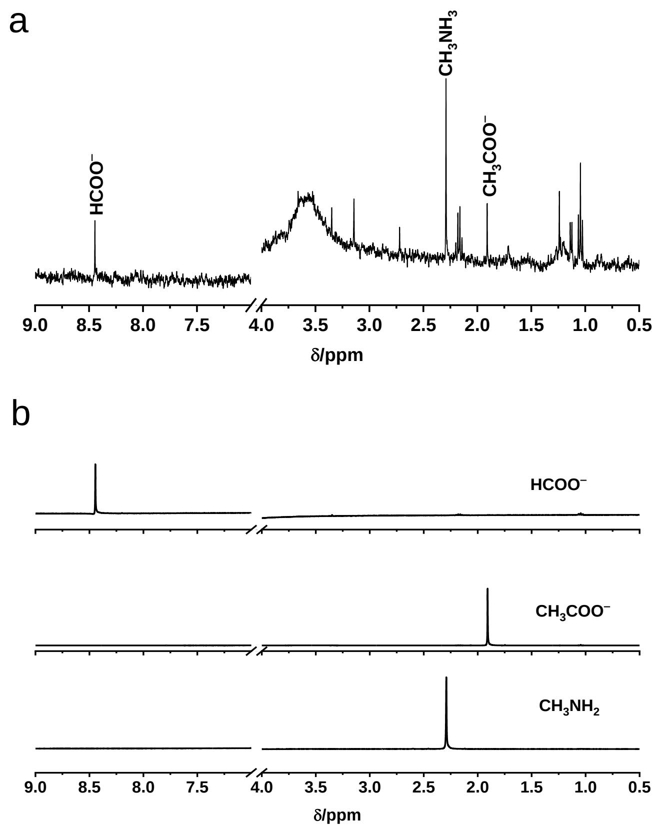

Supplementary Fig. 16. a) ^{1}H-NMR spectrum of products generated by electrolysis of 5 mM CN^- in phosphate buffer (pH=7) on NiS for two weeks. b) ^{1}H-NMR spectra of authentic standards.

pH of product solutions was adjusted to ~12.5 before NMR measurements.

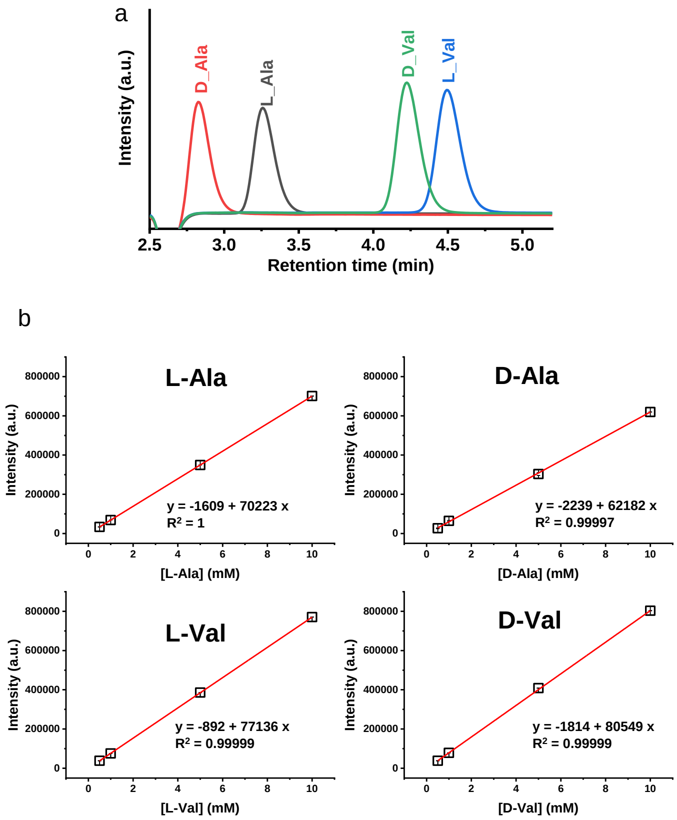

Supplementary Fig. 17. Retention time (a) and calibration curves (b) for L- and D-amino acids detected by UV-HPLC system using a chiral column.

The error bars are the standard deviations associated with the repeated measurements. Abbreviations: Ala: alanine; Val: valine.

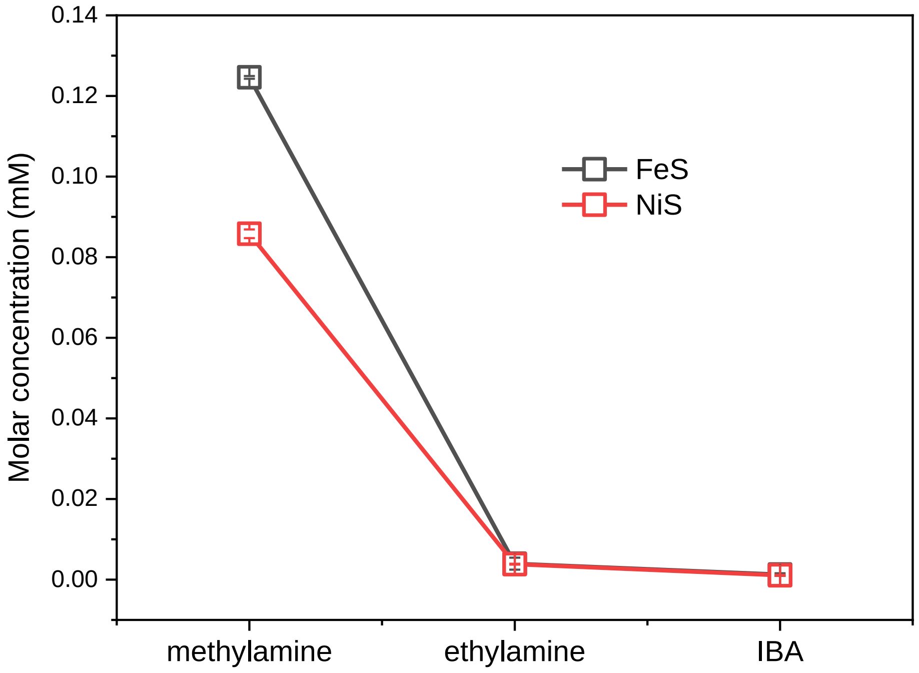

Supplementary Fig. 18. Molar concentrations of primary amines generated in the electrochemical decomposition system of glycine, alanine, and valine, under –0.9 V on FeS (black) and NiS (red) at the initial amino acids concentration of 2 mM.

Methylamine, ethylamine, and isobutylamine (IBA) were generated by electrolysis of glycine, alanine and valine at a substrate concentration of 2 mM for two weeks. Since all these three amino acids generate methylamine, the presented methylamine concentration is the sum of values from all the three amino acid electrolysis experiments operated under the same condition. The error bars are the standard deviations associated with the repeated measurements.

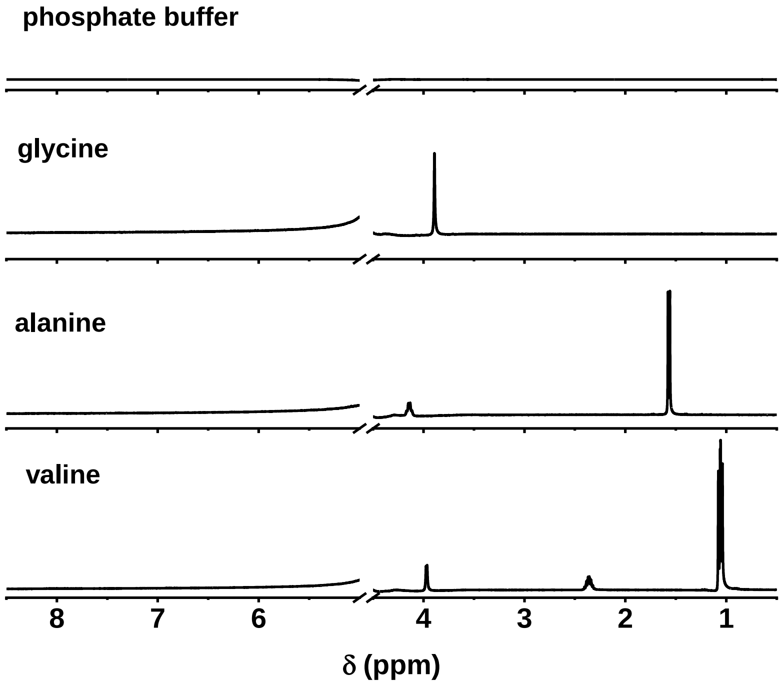

Supplementary Fig. 19 ^{1}H-NMR spectra of starting compounds (glycine, alanine, valine) and phosphate buffer.

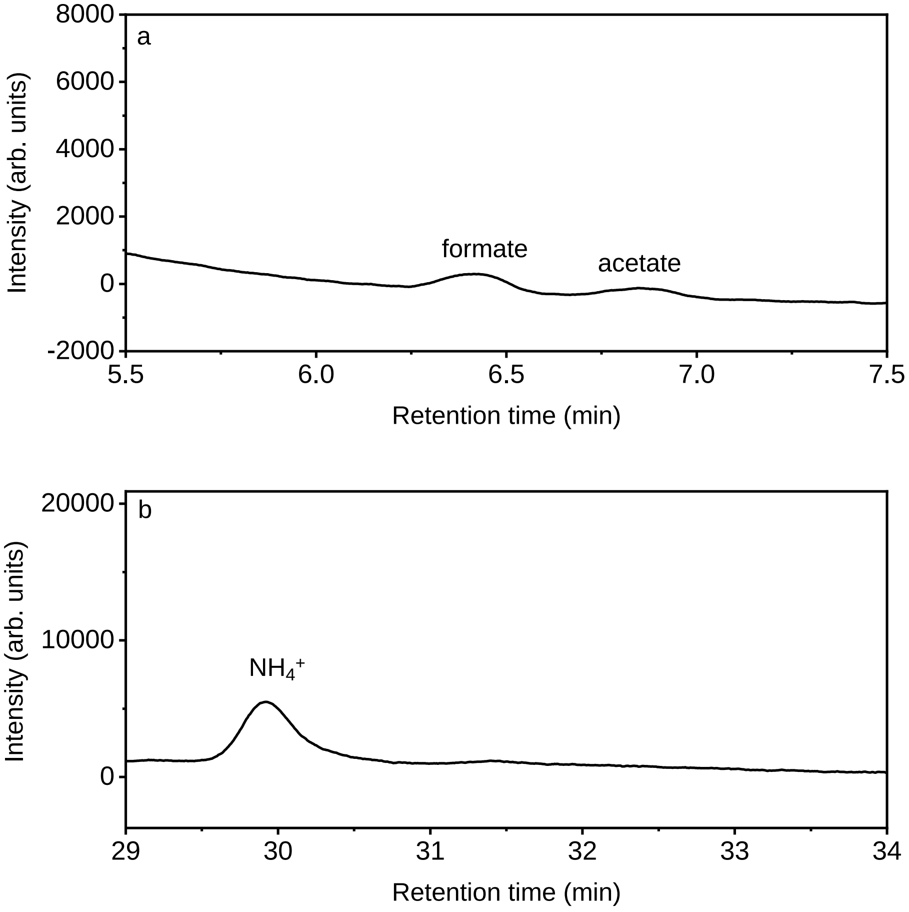

Supplementary Fig. 20 IC (a) and fluorescence (b) chromatograms of a control sample without adding any of the amino acid.

The sample was collected after two-week electrolysis of a phosphate buffered electrolyte (pH 7) at −0.9 V using FeS as a catalyst. Neither hydroxy acids nor primary amines were detected.

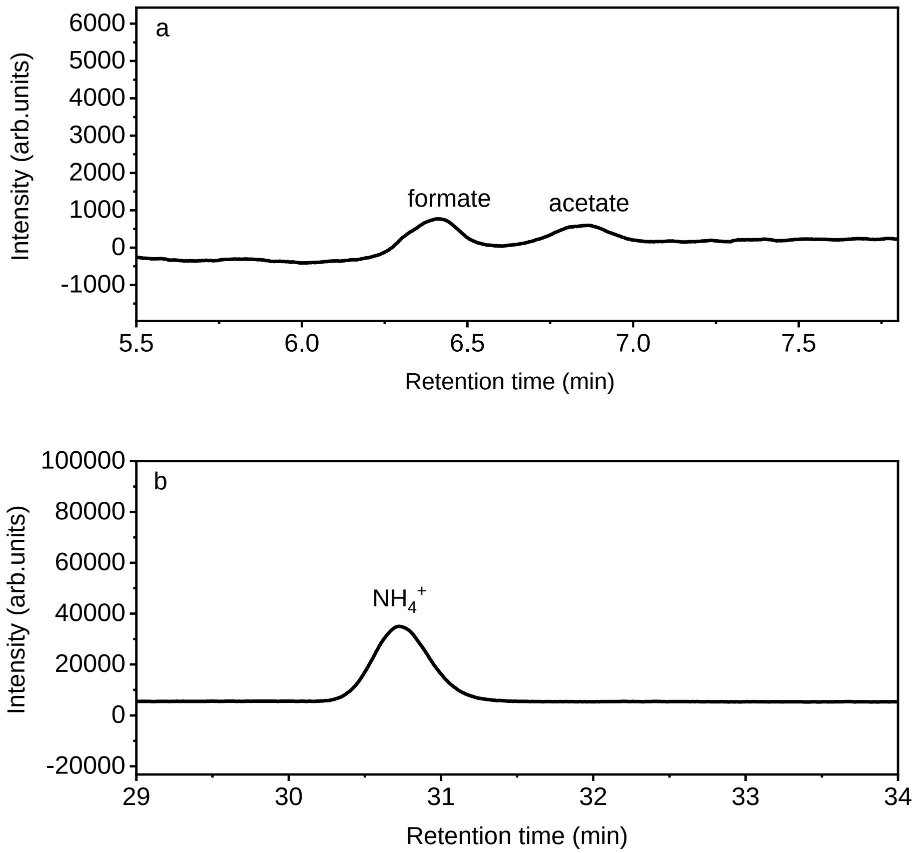

Supplementary Fig. 21 IC (a) and fluorescence (b) chromatograms of a control sample without using any mineral catalyst.

The sample was collected after two-week electrolysis of glycine (20 mM) in a phosphate buffered electrolyte (pH 7) at −0.8 V using a bare carbon paper electrode. Neither glycolate nor methylamine were detected.

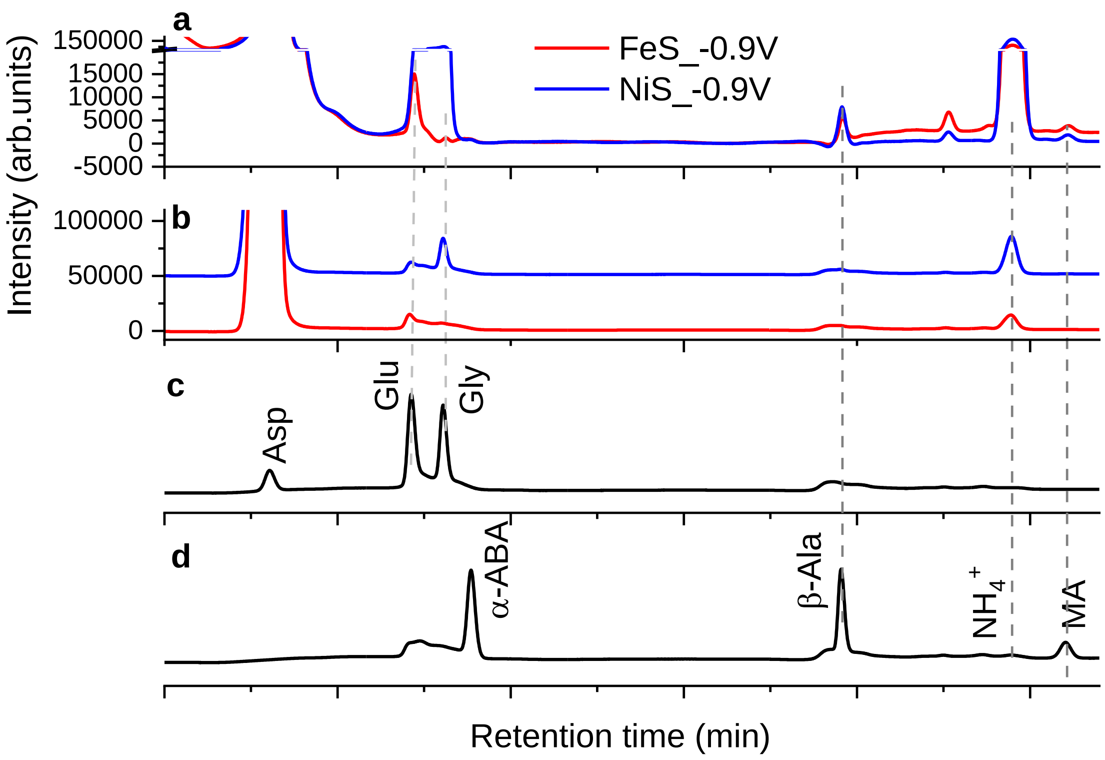

Supplementary Fig. 22 Fluorescence chromatograms of samples generated by electrolysis of aspartic acid.

The samples were collected after two-week electrolysis of aspartic acid (20 mM) in a phosphate buffered electrolyte (pH 7) at −0.9 V using FeS (red) or NiS (blue) as the catalyst. c and d show the chromatograms of authentic standards. In a and c, the injection volumes were 100 µl. In b and d, the injection volumes were 10 µl. Abbreviations: Asp: aspartic acid; Glu: glutamic acid; Gly: glycine; α-ABA: α-aminobutyric acid; β-Ala: β-alanine; MA: methylamine.

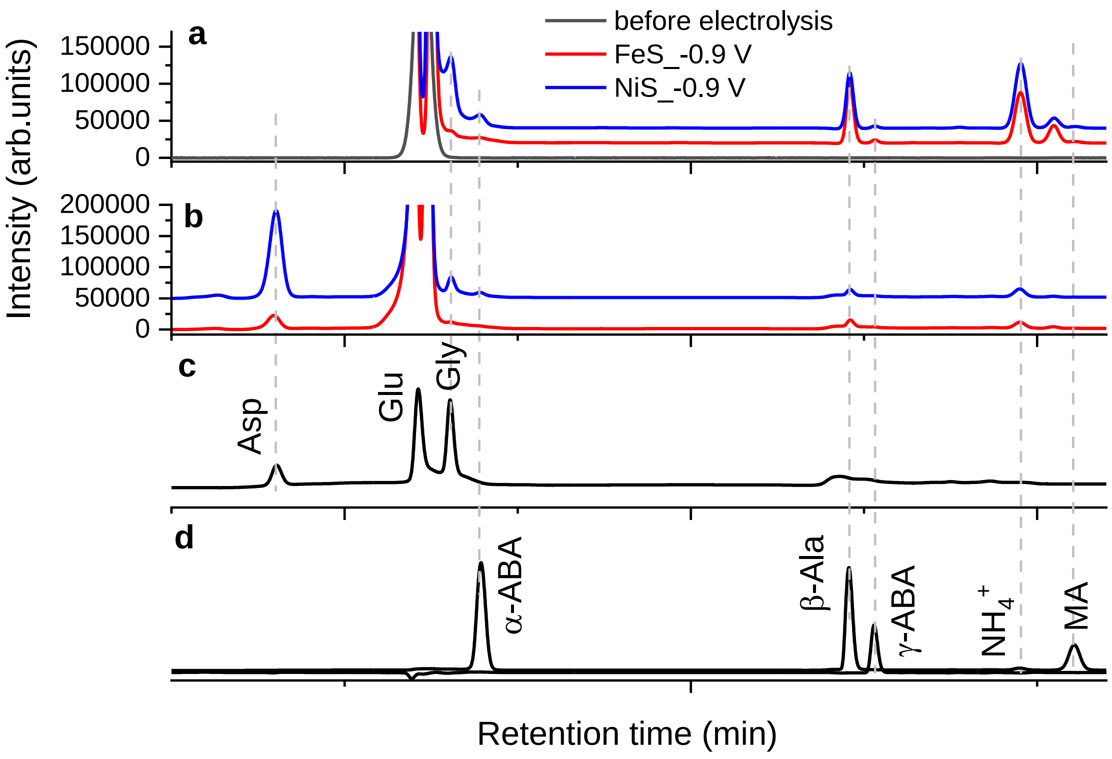

Supplementary Fig. 23 Fluorescence chromatograms of samples generated by electrolysis of glutamic acid.

The samples were collected after two-week electrolysis of Glu (20 mM) in a phosphate buffered electrolyte (pH 7) at −0.9 V using FeS (red) or NiS (blue) as the catalyst. c and d show the chromatograms of authentic standards. In a and d, the injection volumes were 100 µl. In b and c, the injection volumes were 10 µl. Abbreviations: Asp: aspartic acid; Glu: glutamic acid; Gly: glycine; α-ABA: α-aminobutyric acid; β-Ala: β-alanine; γ-ABA: γ-aminobutyric acid; MA: methylamine.

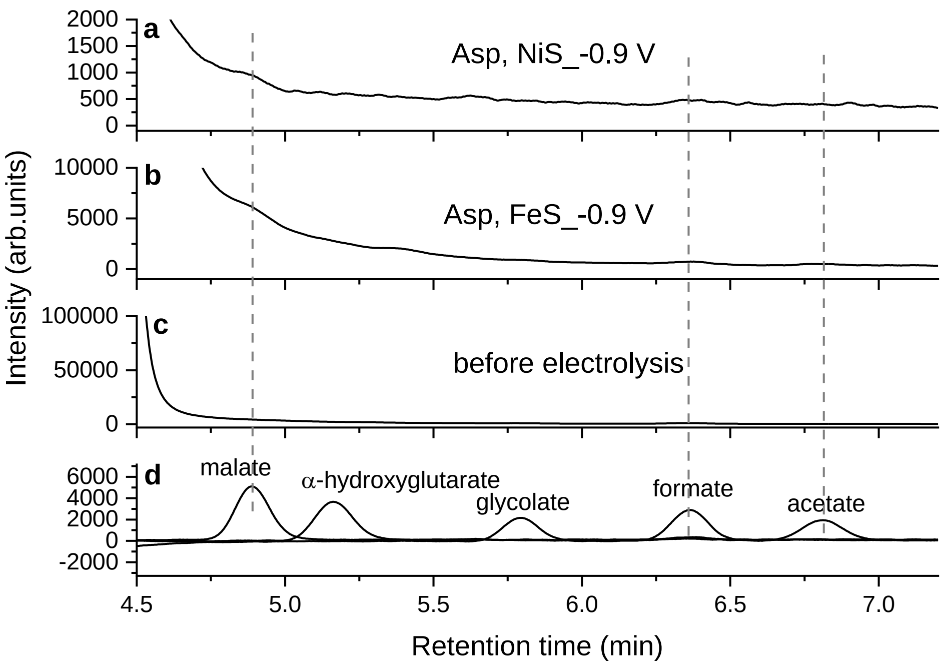

Supplementary Fig. 24 IC chromatograms of samples generated by electrolysis of aspartic acid.

The samples were collected after two-week electrolysis of aspartic acid (20 mM) in a phosphate buffered electrolyte (pH 7) at −0.9 V using NiS (a) or FeS (b) as the catalyst. c shows the chromatogram of the sample before electrolysis. d shows the chromatogram of authentic standards. Tiny peaks of malate and formate were visible.

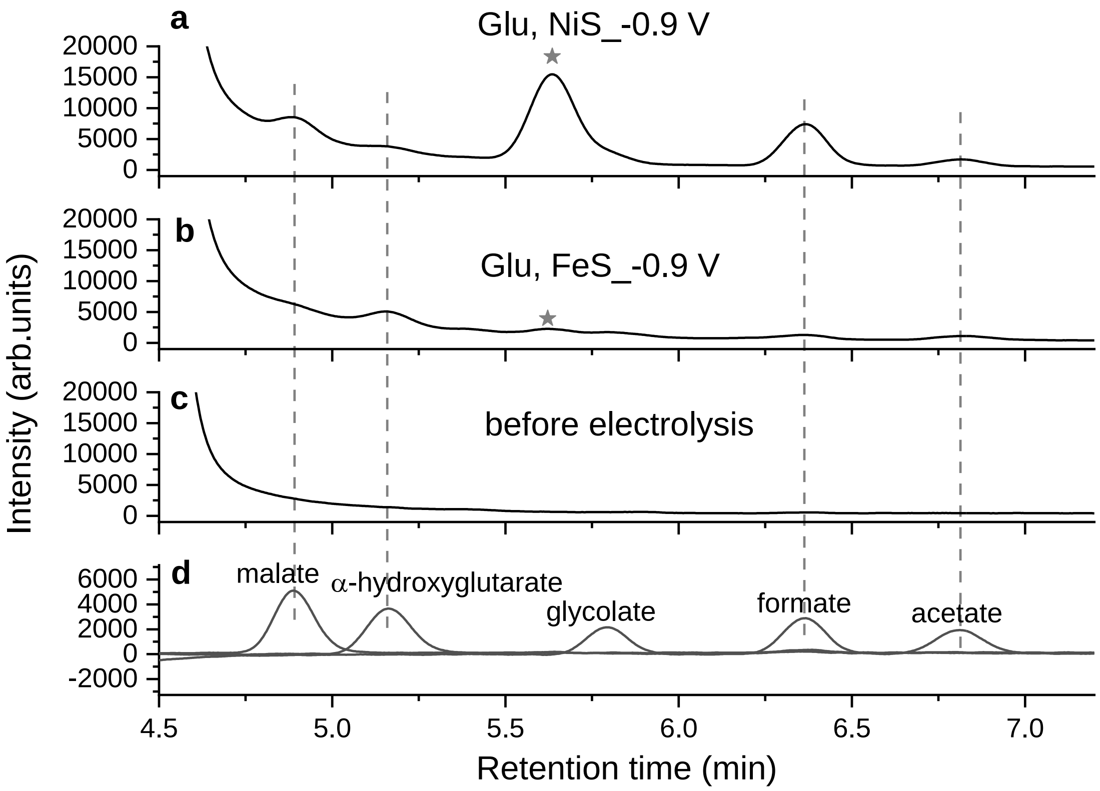

Supplementary Fig. 25 IC chromatograms of samples generated by electrolysis of glutamic acid.

The samples were collected after two-week electrolysis of glutamic acid (20 mM) in a phosphate buffered electrolyte (pH 7) at −0.9 V using NiS (a) or FeS (b) as the catalyst. c shows the chromatogram of the sample before electrolysis. d shows the chromatogram of authentic standards. Malate, α-hydroxyglutarate, formate and acetate were detected as the products.

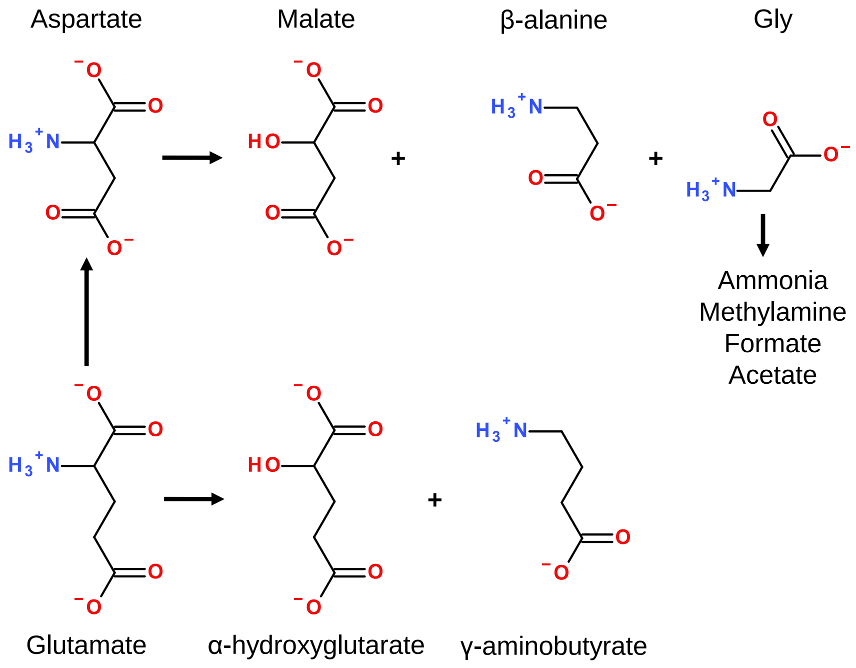

Supplementary Fig. 26 Reaction pathways during electrolysis of aspartate and glutamate. The reaction steps are deduced based on the product analyses in Supplementary Figs. 22-25.

Supplementary Table 1. Summary of the product concentrations after two-week electrolysis of glycine using either FeS or NiS as the catalyst.

Glycine concentration: 20 mM

Catalyst	Potential (V vs SHE)	Concentration/mM				
		ammonia	methylamine	glycolate	formate	acetate
FeS	-0.5	0.62 ± 0.03	0.0069 ± 0.0005	0.0054 ± 0.0002	0.0703±0.0005	0.133±0.005
	-0.6	0.560 ± 0.068	0.0039 ± 0.0006	0.0870 ± 0.0006	0	0
	-0.7	1.11 ± 0.19	0.0083 ± 0.0007	0.0660 ± 0.0001	0	0
	-0.8	2.25 ± 0.20	0.0229 ± 0.0007	0.2578 ± 0.0003	0.0670 ± 0.0002	0.0066 ± 0.0011
	-0.9	1.43 ± 0.15	0.0110 ± 0.0007	0.210 ± 0.002	0.0073 ± 0.0012	0
	-1	0.70 ± 0.06	0.0256 ± 0.0009	0.3998 ± 0.0068	0.110 ± 0.001	0.0210 ± 0.0004
NiS	-0.5	0.62 ± 0.04	0.0087 ± 0.0002	0	0.110 ± 0.002	0.063 ± 0.001
	-0.6	0.53 ± 0.03	0.0040 ± 0.0006	0.0420 ± 0.0012	0.130 ± 0.004	0.012 ± 0.001
	-0.7	0.71 ± 0.05	0.0022 ± 0.0007	0.0031 ± 0.0001	0.046 ± 0.001	0.0180 ± 0.0006
	-0.8	5.29 ± 0.48	0.0120 ± 0.0003	0.0303 ± 0.0011	0.0390 ± 0.0008	0.0064 ± 0.0001
	-0.9	2.80 ± 0.11	0.0092 ± 0.0004	0.1201 ± 0.0007	0.0460 ± 0.0001	0.0170 ± 0.0001
	-1	1.62 ± 0.26	0.0094 ± 0.0004	0.1200 ± 0.0018	0.1102 ± 0.0002	0.0330 ± 0.0008

Supplementary Table 2. Summary of the product concentrations after two-week electrolysis of alanine using either FeS or NiS as the catalyst.

Alanine concentration: 20 mM (10 mM each of L- and D-enantiomers)

Catalyst	Potential (V vs SHE)	concentration/mM					
		ammonia	methylamine	ethylamine	lactate	formate	acetate
FeS	-0.5	3.02 ± 0.14	0.0158 ± 0.0011	0.0045 ± 0.0002	0	0.0136 ± 0.0002	0.0708 ± 0.0016
	-0.6	0.45 ± 0.01	0.0190 ± 0.0005	0.0012 ± 0.00002	0.470 ± 0.002	0.170 ± 0.003	0.2001 ± 0.0004
	-0.7	1.29 ± 0.07	0.0076 ± 0.0001	0.0029 ± 0.00008	0	0.00170 ± 0.00005	0.0049 ± 0.0009
	-0.8	0.93 ± 0.05	0.00790 ± 0.00006	0.0037 ±0.0001	0.1860 ± 0.0006	0.0395 ± 0.0008	0.1897 ± 0.0004
	-0.9	3.12 ± 0.12	0.0148 ± 0.0005	0.0046 ± 0.0002	0	0.0456 ± 0.0006	0.3588 ± 0.0201
	-1	0.67 ± 0.02	0.0037 ± 0.00003	0.0067 ±0.0002	0.526 ± 0.002	0.0710 ± 0.00001	0.2680 ± 0.0075
NiS	-0.5	1.71 ± 0.06	0.0074±0.0002	0.0038 ± 0.0001	0.0202 ± 0.0037	0.0565 ± 0.0020	1.175±0.014
	-0.6	0.88 ± 0.03	0.0047 ± 0.0001	0.0033 ± 0.0001	0	0.0223 ± 0.0013	0.3404 ± 0.0008
	-0.7	1.13 ± 0.03	0.0053 ± 0.0002	0.0035 ± 0.0002	0.0170 ± 0.0001	0.0458 ± 0.0042	0.6853 ± 0.0132
	-0.8	0.91 ± 0.06	0.0074 ± 0.00007	0.0040 ± 0.0002	0.0180 ± 0.0005	0.0734 ± 0.0011	1.0171 ± 0.0201
	-0.9	0.25 ± 0.02	0.0052 ± 0.0002	0.0032 ± 0.0002	0.1360 ± 0.0002	0.164 ± 0.011	2.0152 ± 0.0631
	-1	0.786 ± 0.011	0.0040 ± 0.0001	0.0045 ± 0.0001	0.0717 ± 0.0019	0.140 ± 0.008	2.045 ± 0.091

Supplementary Table 3. Summary of the product concentrations after two-week electrolysis of valine using either FeS or NiS as the catalyst.

Valine concentration: 20 mM (10 mM each of L- and D-enantiomers). Abbreviations: IBA: isobutylamine; HMA: 2-hydroxy-3-methylbutyrate

Catalyst	Potential (V vs SHE)	concentration/mM						
		ammonia	methylamine	IBA	glycolate	formate	acetate	HMA
FeS	-0.5	1.10 ± 0.04	0.0144 ± 0.0004	0	0.0084 ± 0.0001	0.061 ± 0.00007	0	0.2380 ± 0.0012
	-0.6	0.40 ± 0.03	0	0	0	0.0017 ± 0.00002	0.00078 ± 0.00001	0
	-0.7	0.41 ± 0.02	0.0027 ± 0.0001	0	0.0251 ± 0.0001	0.0017 ± 0.0004	0	0.093 ± 0.004
	-0.8	0.58 ± 0.03	0.0042 ± 0.0001	0.0011 ± 0.0003	0	0.0019 ±0.0006	0	0.00087 ± 0.00002
	-0.9	1.15 ± 0.09	0.0014 ± 0.0001	0.0045 ± 0.0002	0.0861 ± 0.0003	0.094 ± 0.006	0.130 ± 0.002	0.310 ± 0.043
	-1	0.67 ± 0.0003	0.0039 ± 0.00002	0.0039 ± 0.00005	0.0910 ± 0.0003	0.210 ± 0.006	0.361 ± 0.006	0.260 ± 0.003
NiS	-0.5	0.42 ± 0.01	0	0	0	0.0356 ± 0.00007	0.01000 ± 0.00009	0
	-0.6	0.51 ± 0.02	0.0011 ± 0.0001	0.0016 ± 0.00007	0	0.0071 ± 0.0009	0	0
	-0.7	0.61 ± 0.02	0.0011 ± 0.00005	0.0020 ± 0.00004	0.0151 ± 0.0001	0.032 ± 0.001	0.016 ± 0.002	0
	-0.8	0.42 ± 0.01	0	0.002 ± 0.0005	0.0046 ± 0.0002	0.063 ± 0.001	0.0401 ± 0.0001	0
	-0.9	0.530 ± 0.006	0	0	0.0301 ± 0.0001	0.280 ± 0.008	0.420 ± 0.026	0
	-1	0.35 ± 0.01	0.0024 ± 0.00006	0.0026 ± 0.00002	0.03202 ± 0.00001	0.2270 ± 0.0009	0.450 ± 0.002	0

Supplementary Table 4. Abundances of amino acids in various CM and CR chondrites.

Amino acid abundances/nmol g^{-1} of meteorite	Glycine	Alanine	Valine	Ref.
Asuka 12236 (CM2.9)	160 ± 5	58 ± 4	12.4 ± 1.3	1
Paris (CM2.7)	110 ± 5	18.8 ± 0.9	10.1 ± 0.3	
Murchison (CM2.5)	40 ± 3	5.2 ± 0.3	3.35 ± 0.12	
GRO 95577 (CR2.0)	3.7 ± 1	0.91 ± 0.24	0.15 ± 0.02	2
MIL 090001 (CR 2.4)	3.46 ± 0.88	1.99 ± 0.53	0.2 ± 0.03	
MIL 090657 (CR2.7)	275 ± 23	527 ± 38	31.5 ± 4.7	
GRA 95229 (CR2.7)	770 ± 5	1138 ± 37	100.7 ± 3.1	
EET 92042 (CR2.8)	726 ± 205	914 ± 188	126.9 ± 7.9	
QUE 99177 (CR2.8)	188 ± 45	80.1 ± 11.81	62.4 ± 3	

Supplementary Table 5. Abundances of amines in various CM and CR chondrites.
Errors were not included if not reported. For the abundances that were not reported, the data were denoted as "-" symbols.

Amine abundances/nmol g^{-1} of meteorite	Methylamine	Ethylamine	Isobutylamine	Ref.
ALH 83100 (CM1/2)	29.6 ± 3.2	7.8 ± 1.1	0.4 ± 0.03	3
LEW 90500 (CM2)	101.1 ± 2.1	42.3 ± 1.2	2.8 ± 0.6	3
Murchison (CM2)	85.1 ± 7.8	24.1 ± 2.8	1.6 ± 0.2	4
LON 94101 (CM2)	33.7 ± 1.5	10.5 ± 0.5	0.8 ± 0.01	3
Orgueil (CI1)	331.5 ± 0.5	27.3 ± 2.4	< 0.7	4
LAP 02342 (CR2)	261 ± 27.1	97.5 ± 6.5	8.2 ± 0.6	3
GRO 95577 (CR2.0)	43.8	-	-	2
MIL 090001 (CR2.4)	24.4 ± 2.9	11.6 ± 1.3	< 0.01	
GRA 95229(CR2.7)	493.2 ± 12.5	186 ± 6	13.9 ± 0.5	
MIL 090657 (CR2.7)	394.5 ± 18.8	73.6 ± 4.6	14.1 ± 0.8	
EET 92042 (CR2.8)	27.7	17.9	-	
QUE 99177 (CR2.8)	93.1	29.8	-	

Supplementary Table 6. Abundances of hydroxy acids in various CM and CR chondrites.

Errors were estimated to be 5% for the date sourced from ref. 5. In ref. 2, errors for GRO 95577, GRA 95229, EET 92042, and QUE 99177 were not reported. For the abundances that were not reported, the data were denoted as "-" symbols. * HMA abundance was reported in another paper [5]. Abbreviation: HMA: 2-hydroxy-3-methylbutyrate.

Hydroxy acid abundances/nmol g^{-1} of meteorite	glycolate	lactate	HMA	Ref.
Murchison (CM2)	65 ± 3.2	84.2 ± 4.2	36.9 ± 1.8	5
LAP 02342 (CR2)	326.2 ± 16.3	208.5 ± 10.4	3.1 ± 0.1	5
GRO 95577 (CR2.0)	1084.1	484.9	-	2
MIL 090001 (CR2.4)	40.0 ± 5.1	92.8 ± 10.3	-	
GRA 95229 (CR2.7)	66.7	34.6	3*	
MIL 090657 (CR2.7)	52.6 ± 4.8	33.6 ± 2.3	-	
EET 92042 (CR2.8)	473.1	131.8	-	
QUE 99177 (CR2.8)	156.8	174	-	

Supplementary Table 7. Abundance ratios of methylamine/glycine ([MA]/[Gly]), glycolate/glycine ([GA]/[Gly]), ethylamine/alanine ([EA]/[Ala]), lactate/alanine ([LA]/[Ala]), isobutylamine/valine ([IBA]/[Val]), 2-hydroxy-3-methylbutyrate/valine ([HMA]/[Val]) in different CR chondrites based on the data listed in Supplementary Tables 4-6.

	[MA]/[Gly]	[GA]/[Gly]	[EA]/[Ala]	[LA]/[Ala]	[IBA]/[Val]	[HMA]/[Val]
GRO 95577 (CR2.0)	11.83 ± 3.20	293 ± 79	0	532.85 ± 140.53	--	--
MIL 090001 (CR2.4)	7.05 ± 1.97	11.56 ± 3.29	5.83 ±1.68	46.63 ± 13.45	0.05 ± 0.0075	--
MIL 090657 (CR2.7)	1.43 ± 0.14	0.19 ± 0.02	0.139 ± 0.013	0.063 ± 0.006	0.44 ± 0.07	--
GRA 95229 (CR2.7)	0.64 ± 0.01	0.086 ± 0.0005	0.163 ± 0.007	0.03 ± 0.0009	0.138 ± 0.006	0.015
EET 92042 (CR2.8)	0.038 ± 0.01	0.65 ± 0.18	0.019 ± 0.004	0.14 ± 0.03	--	--

QUE 99177 (CR2.8)	0.49 ± 0.12	0.83 ± 0.20	0.37 ± 0.05	2.17 ± 0.32	--	--

Supplementary Table 8. Molar ratios of glycolate/methylamine, lactate/ethylamine, and 2-hydroxy-3-methylbutyrate/isobutylamine ([HMA]/[IBA]) in five different CR chondrites (GRO 95577 (CR2.0), MIL 090001 (CR2.4), GRA 95229 (CR2.7), MIL090657 (CR2.7), and QUE 99177 (CR2.8)) calculated based on reported abundances in literatures as tabulated in Supplementary Tables 5-6.

	[Glycolate]/[Methylamine]	[Lactate]/[Ethylamine]	[HMA]/[IBA]
GRO 95577 (CR2.0)	24.75	--	--
MIL 090001 (CR2.4)	1.64 ± 0.28	8.00 ± 1.26	--
MIL 090657 (CR2.7)	0.13 ± 0.01	0.46 ± 0.04	--
GRA 95229 (CR2.7)	0.14 ± 0.003	0.19 ± 0.006	0.22
EET 92042 (CR2.8)	17.08	7.36	--
QUE 99177 (CR2.8)	1.68	16.73	--

Supplementary References

1. Glavin DP, *et al.* Abundant extraterrestrial amino acids in the primitive CM carbonaceous chondrite Asuka 12236. *Meteorit Planet Sci* **55**, 1979-2006 (2020).

2. Aponte JC, *et al.* Analysis of amino acids, hydroxy acids, and amines in CR chondrites. *Meteorit Planet Sci* **55**, 2422-2439 (2020).

3. Aponte JC, McLain HL, Dworkin JP, Elsila JE. Aliphatic amines in Antarctic CR2, CM2, and CM1/2 carbonaceous chondrites. *Geochim Cosmochim Acta* **189**, 296-311 (2016).

4. Aponte JC, Dworkin JP, Elsila JE. Indigenous aliphatic amines in the aqueously altered Orgueil meteorite. *Meteorit Planet Sci* **50**, 1733-1749 (2015).

5. Pizzarello S, Wang Y, Chaban GM. A comparative study of the hydroxy acids from the Murchison, GRA 95229 and LAP 02342 meteorites. *Geochim Cosmochim Acta* **74**, 6206-6217 (2010).
